# Dobutamine stress cardiac MRI reliably predicts significant coronary disease in renal transplant candidates

**DOI:** 10.1186/1532-429X-16-S1-P181

**Published:** 2014-01-16

**Authors:** Anthony D Pisaniello, Benjamin K Dundon, Murilo Maia, Karen S Teo, Stephen G Worthley, Kym Bannister, Randall Faull, Patrick T Coates, Graeme R Russ, Matthew I Worthley

**Affiliations:** 1 Cardiovascular Investigational Unit, Royal Adelaide Hospital, Adelaide, South Australia, Australia; 2 Renal and Transplantation Service, Royal Adelaide Hospital, Adelaide, South Australia, Australia; 3 Cardiology Unit, Monash Medical Centre, Melbourne, Victoria, Australia

## Background

Coronary artery disease (CAD) accounts for half of all deaths in patients with end stage kidney disease (ESKD) who have undergone renal transplantation. In patients with ESKD, "angiographically" significant CAD may be asymptomatic. Identification of these patients, with subsequent revascularisation, may reduce the prevalence of adverse cardiovascular events in the peri-transplant and post-transplant period. We aimed to evaluate the effectiveness of diagnostic (>85% maximum age-predicted heart rate) dobutamine stress cardiac magnetic resonance (DSCMR) imaging in identifying "angiographically" significant CAD in asymptomatic patients.

## Methods

Over a five year period, 62 high-risk patients were referred to this program by the nephrology team. All of these subjects had an invasive coronary angiogram (ICA). These subjects had ESKD and were being considered for renal transplantation. 42 (68%) were male. All had at least one traditional cardiovascular risk factor. 58 (94%) were on renal replacement therapy. Of the 62 enrolled patients, 43 (69%) had a diagnostic DSCMR followed by an ICA. ICA reporters were blinded to results of DSCMR and vice-versa. 19 patients (31%) were excluded from the analysis due to non-diagnostic DSCMR scans. The most common reasons for a nondiagnostic DSCMR included insufficient augmentation in heart rate with dobutamine stress (in 8 patients) and claustrophobia (in 5 patients). Significant CAD was defined on ICA as a coronary stenosis of ≥ 70%.

## Results

Of the 43 included patients, 12 (28%) had significant CAD, and all of these patients had evidence of inducible myocardial ischaemia on DSCMR. 3 (7.0%) patients had false positive DSCMR scans. There were no false negative scans. Of the 19 patients with non-diagnostic CMR scans, 7 patients had significant CAD on ICA. In this cohort studied with a diagnostic DSCMR: sensitivity = 100%, specificity = 90%, positive predictive value = 80%, negative predictive value = 100%. Over this period, 26 patients have undergone successful renal transplantation and 8 patients have died (none of which had undergone renal transplantation).

## Conclusions

When feasible, a diagnostic DSCMR can reliably detect "angiographically" significant CAD in patients with ESKD being considered for renal transplantation.

## Funding

Nothing to declare.

